# Preventable maternal mortality: Geographic/rural-urban differences and associated factors from the population-based maternal mortality surveillance system in China

**DOI:** 10.1186/1471-2458-11-243

**Published:** 2011-04-19

**Authors:** Juan Liang, Li Dai, Jun Zhu, Xiaohong Li, Weiyue Zeng, He Wang, Qi Li, Mingrong Li, Rong Zhou, Yanping Wang

**Affiliations:** 1 National Office for Maternal and Child Health Surveillance, West China Second University Hospital, Sichuan University, No 17, section 3, Ren Min Nan Lu, Chengdu, Sichuan, China; 2 National Center for Birth Defects Monitoring, West China Second University Hospital, Sichuan University, No 17, section 3, Ren Min Nan Lu, Chengdu, Sichuan, China; 3 Obstetric and Gynecologic Department, West China Second University Hospital, Sichuan University, No 17, section 3, Ren Min Nan Lu, Chengdu, Sichuan, China

## Abstract

**Background:**

Most maternal deaths in developing countries can be prevented. China is among the 13 countries with the most maternal deaths; however, there has been a marked decrease in the maternal mortality ratio (MMR) over the last 3 decades. China's reduction in the MMR has contributed significantly to the global decline of the MMR. This study examined the geographic and rural-urban differences, time trends and related factors in preventable maternal deaths in China during 1996-2005, with the aim of providing reliable evidence for effective interventions.

**Methods:**

Data were retrieved from the population-based maternal mortality surveillance system in China. Each death was reviewed by three committees to determine whether it was avoidable. The preventable maternal mortality ratio (PMMR), the ratios of PMMR (risk ratio, RR) and 95% confidence intervals (CI) were used to analyze regional disparities (coastal, inland and remote regions) and rural-urban variations. Time trends in the MMR, along with underlying causes and associated factors of death, were also analysed.

**Results:**

Overall, 86.1% of maternal mortality was preventable. The RR of preventable maternal mortality adjusted by region was 2.79 (95% CI 2.42-3.21) and 2.38 (95% CI: 2.01-2.81) in rural areas compared to urban areas during the 1996-2000 and 2001-2005 periods, respectively. Meanwhile, the RR was the highest in remote areas, which was 4.80(95%CI: 4.10-5.61) and 4.74(95%CI: 3.86-5.83) times as much as that of coastal areas. Obstetric haemorrhage accounted for over 50% of preventable deaths during the 2001-2005 period. Insufficient information about pregnancy among women in remote areas and out-of-date knowledge and skills of health professionals and substandard obstetric services in coastal regions were the factors frequently associated with MMR.

**Conclusions:**

Preventable maternal mortality and the distribution of its associated factors in China revealed obvious regional differences. The PMMR was higher in underdeveloped regions. In future interventions in remote and inland areas, more emphasis should be placed on improving women's ability to utilize healthcare services, enhancing the service capability of health institutions, and increasing the accessibility of obstetric services. These approaches will effectively lower PMMR in those regions and narrow the gap among the different regions.

## Background

Since the United Nations proposed a 75% reduction in the global maternal mortality ratio (MMR) by 2015 over the 1990 figure [1], governments worldwide have defined their own goals and strategies for MMR reduction accordingly. Despite the endeavours made thus far, the global MMR in 2005 was not much different from that in 1990. The MMR even remained the same in some countries/regions, such as sub-Saharan Africa, or only slightly decreased, as in South Asia [2,3]. Thus, it is very challenging to reduce the global MMR to the expected level and to achieve the Millennium Development Goals by 2015.

Nevertheless, many studies have shown that further reductions in maternal mortality can still be made in developing countries by improving the maternal health system, initiating special interventions, and utilizing other approaches such as public health education. It was estimated that over 342,900 (99%) maternal deaths occurred in developing countries [4], and 37-90% of them could have been prevented [5]. Obviously, women in developing countries/areas have a higher risk of dying from pregnancy complications. One study reported that 1 in 16 sub-Saharan women, 1 in 94 Asian women and 1 in 4000 European women died from pregnancy-related complications [6]. Studies also documented a remarkable racial/ethnic variation in the MMR. In the United States, African-American women had higher MMR than white women [7], while in Germany [8] and the Netherlands[9], immigrants had higher MMR than the natives. To minimize the regional/racial/ethnic differences in MMR, Germany improved access and quality of obstetric services for non-native women [8], Chile promoted education on health and healthcare access [10], and Sri Lanka controlled infection [11]. In developed and developing countries, these approaches have been proven to be feasible and effective in minimizing the socioeconomic gap and the negative effects of traditional culture on reproductive health, thus eventually lowering the MMR.

Over the past two decades, China has experienced remarkable economic growth. The overall health status of the Chinese population has improved remarkably with the increased government investment in health, although the increase is not proportional to the country's tremendous economic growth [12]. However, there has been a great gap in socioeconomic development between rural and urban areas and among different geographic regions (coastal, inland and remote regions), which in turn affects China's medical care [13]. The distribution of maternal deaths and associated factors also varies markedly among different regions within this country. Several researchers proposed that health inequality in China could be attributed to the unbalanced socioeconomic development [13-15]. How to narrow the rural-urban and regional gap in MMR is a challenge faced by the Chinese government. Health professionals, policy makers, and even the public are concerned about whether and how the MMR in China can be reduced. Based on the nationwide maternal mortality surveillance data from 1996 to 2005, the present study examined the rural-urban and geographic differences in preventable maternal mortality, analysed the time trends and associated factors, and discussed strategies and methods to reduce maternal death.

## Methods

### NMMSS in China

Data used in this study were obtained from the National Maternal Mortality Surveillance System (NMMSS), a well-established population-based maternal death registry system set up by the Ministry of Health of China in 1989. This system now includes 176 counties/districts as the basic sampling unit, covering a population of 60 million within mainland China. The selected counties/districts can be classified into urban (97) or rural areas (79) according to the women's residence, or into coastal (62), inland (54), and remote regions (60) according to their geographic locations and socioeconomic status [16,17]. The data collected by the system are included in the National Health Statistics Yearbook [18].

### Data collection

The data collection procedures have been documented previously [13,19]. In short, the numbers of maternal mortalities and live births were collected monthly by trained village doctors and reported to township maternal and child healthcare professionals, who examined and summarized data using a standardized quarterly report. The data were then sent to the county and provincial maternal and child healthcare institutions to be reviewed, corrected and verified. Ultimately, the data were submitted to the National Office for Maternal and Child Health Surveillance, where an expert panel was responsible for the final verification, maternal death review and data analysis.

### Data quality management

To guarantee the accuracy of surveillance data, the NMMSS employed a set of strict underreporting investigation methods in addition to the routine collection of data on live births and maternal mortality. Every year, prior to the annual report, the surveillance systems at the county/district, provincial and state levels will conduct independent retrospective quality investigations of data submitted by counties/districts. The investigators must visit departments related to maternal mortality surveillance, such as the Vital Statistical System, Household Registration Department of the Ministry of Public Security, healthcare institutions, and departments of family planning and crematories to verify the number of live births, maternal mortality and causes of death and make prompt corrections to underreporting or misdiagnoses of death cause.

### Investigation of maternal deaths

To collect information for maternal death review, an investigation was conducted by at least one specialty trained obstetrician from the county/district maternal and child health hospital. For deaths inside the hospital, hospital records and other related information were collected by document reviewing or personal interviewing. For deaths outside the hospital, information was collected by household visit. This sort of investigation focused on personal/family background, overall situation during pregnancy, onset of any disease and corresponding treatment, course of delivery, the date of termination of pregnancy, and the date of maternal death. Subjects of investigation were the husband, family members and relatives, midwife, obstetrician, nursing staff and others close to the deceased woman. Upon completion of the investigation, the investigation form, medical examiner reports, medical records, and autopsy reports for each case were submitted to the review committee at the county/district level of the surveillance area.

### Maternal death review

A death was considered preventable ''if the death may have been averted by one or more changes in the healthcare system related to clinical care, facility infrastructure, public health infrastructure and/or patient factors.'' For each case classified as potentially preventable, a determination was made regarding the ways in which the death might have been averted [20].

According to the World Health Organization (WHO) recommendation [21], each case of maternal death was reviewed by the review committees at the county/district, provincial, and state levels. After receiving the mortality report, the committee at the county/district level conducted the preliminary review: (1) to check the completeness and accuracy of the report and request additional information for the report if needed and (2) to determine causes of death, identify preventable areas and associated factors, and suggest interventions. The review committees at the provincial and state level re-examined the results submitted by the subordinate committee, undertook the joint review for contentious cases, and made the final decision based on the majority of opinions. Causes of death were encoded in accordance with ICD-10[22].

The review committee identified three areas in which different actions might have prevented the deaths: individual/family, health institutions, and the social departments. Each of these areas involved four factors: knowledge/skills, attitude, resources, and management (Table 1). In detail, the factor would be regarded as the knowledge of the individual when the woman did not receive prenatal examination due to her unawareness of its necessity, had home delivery due to her ignorance of its risk, or did not receive any treatment for medical conditions diagnosed before pregnancy because she did not know the conditions could have been aggregated by pregnancy. Factors would be grouped into the attitude of the individual when the woman or her family refused necessary treatment or did not adhere to medical advice for personal reasons. Factors would be classified as the resources of the individual/family when the woman failed to seek care timely because of poverty or inconvenient transport. Factors would belong to the knowledge/skills of the health professionals in health institutions when the professionals failed to identify and to solve problems related to the pregnancy, delivery and puerperium of the woman. Factors would be counted as the attitude of the health professionals in health institutions when they lacked a sense of responsibility, neglected necessary health education and were reluctant for referral. Factors would be considered as the resources of the health institutions when they were short of first-aid drugs, equipment, blood supply or technicians. Factors would be considered as the management of the health institutions when the equipment for first aid failed to function, a sound diagnosis and treatment system was absent, or a system for treatment of critically ill and emergency cases was lacking. Factors would be grouped into the social departments when the departments of transportation, family planning, and the working committee on women and children were involved.

**Table 1:** The factors associated with preventable maternal deaths indentified by review committee

	Individual/family	Health institutions	Social departments
Knowledge/skills			

Attitude			

Resources			

Management			

### Statistical analysis

Considering that maternal death is an event of extremely small probability, we combined the five-year data to get a more stable MMR. The MMR ratio (risk ratio, RR) and its 95% confidence interval (95% CI) were used to measure the rural-urban and regional disparities in preventable maternal mortality ratio (PMMR) and cause-specific PMMR [23]. When using RR to compare the rural-urban and regional disparities, the PMMR in urban areas and coastal areas were set as the reference group. Cochran-Mantel-Haensel (CMH) was used to calculate the RR and its 95% CI was adjusted by the factor of residential areas or geographic areas and stratified by the time periods. When different reference groups were set, the RR in this study was used not only to assess the relative risks but also to predict the number of deaths that would be reduced. Time trends in MMR were tested using the Cohran-Armitage trend test (SAS version 9.0; SAS Institute, Carey, NC, USA) [24]. The standard chi-square test was used to compare the distributions of factors associated with maternal death. The statistical significance level for α was set at 0.05.

## Results

### Preventable maternal mortality during 1996-2005

As shown in Table 2, 2949 maternal deaths were identified by NMMSS during the 1996-2005 period, among which 2540 cases (86.1%) were determined to be preventable. The overall PMMR varied significantly by time period, residence area and geographic region. Generally, a decreasing trend on PMMR in China was detected with the PMMR during the 1996-2000 period, which was higher than that during the 2001-2005 period. Higher PMMR was observed in rural areas and remote regions than in urban areas and costal region, respectively. The proportion of preventable deaths was also higher in rural than in urban areas, with the highest in remote rural areas. Compared with the years from 1996 to 2000, the proportions of preventable deaths in both urban and rural areas during the 2001-2005 period decreased, with the exception of urban areas situated inland (Table 2). An interesting phenomenon is that the proportion of preventable maternal deaths increased in this order: costal, inland and remote areas. The rural over urban PMMR ratios within specific stratifications and adjusted ones revealed a huge gap between rural and urban areas (Table 2). Although the PMMR for both urban and rural areas had decreased over time, the adjusted rural over urban RR was still as high as 2.38 (95% CI:2.01-2.81) between 2001 and 2005.

**Table 2:** Rural-urban disparities of preventable maternal mortality in China, 1996-2000 and 2001-2005

	Urban				Rural				**RR **^**b **^**(95% CI)**
			
	Live births	Deaths	**PMMR**^**a**^	Proportion (%)	Live births	Deaths	**PMMR**^**a**^	Proportion (%)	
1996-2000									
Coastal	597590	90	15.06	65.7	542370	152	28.03	83.52	1.86 (1.43-2.42)
Inland	426794	102	23.90	75.0	810690	553	68.21	91.56	2.85 (2.31-3.53)
Remote	134319	55	40.95	80.9	373172	560	150.06	97.05	3.66 (2.78-4.83)
Total	1158703	247	21.32	72.4	1726232	1265	73.28	92.81	2.79 (2.42-3.21)*
									
2001-2005									
Coastal	550516	57	10.35	59.4	482010	82	17.01	73.21	1.64 (1.17-2.30)
Inland	374354	77	20.57	77.0	850264	450	52.92	84.75	2.57 (2.02-3.28)
Remote	131795	44	33.39	75.9	358094	318	88.80	91.38	2.66 (1.94-3.65)
Total	1056665	178	16.85**	70.1	1690368	850	50.28**	85.77	2.38 (2.01-2.81)*

*Note*. CI = confidence interval.

^a^PMMR was calculated as the number of preventable maternal deaths per 100 000 live births.

^b^RR = risk ratio

*Adjusted for regions using CMH

**P < 0.05

Similarly, significant regional disparities were identified during the 1996-2005 period. The PMMRs and the adjusted RR revealed that women in remote areas have the highest risk of dying from pregnancy, followed by women who live in inland (Table 3). Although PMMRs and proportions of preventable deaths by geographical classification declined significantly during the years from 2001 to 2005, regional gaps in preventable maternal deaths tended to increase as shown by the RR indicators. Within the urban stratification, the RR of remote to coastal and the RR of inland to coastal during the 2001-2005 were higher than those during the 1996-2000 period. Within the rural stratification, higher inland to coastal RR was observed during the 2001-2005 period (Table 3).

**Table 3:** Comparison of preventable maternal mortality ratio among different regions, China, 1996-2000 and 2001-2005

Region	**RR**^**a**^**(95% CI)**			Preventable proportion (%)
		
	Urban-Urban	Rural-Rural	Adjusted*	
1996-2000				
Coastal	1	1	1	75.86
Inland	1.59(1.20-2.11)	2.43(2.03-2.91)	2.19(1.88-2.54)	88.51
Remote	2.72(1.95-3.80)	5.35(4.48-6.41)	4.80(4.10-5.61)	95.35
				
2001-2005				
Coastal	1	1	1	66.83
Inland	1.99(1.41-2.80)	3.11(2.46-3.94)	2.77(2.28-3.36)	83.52
Remote	3.22(2.18-4.78)	5.22(4.09-6.65)	4.74(3.86-5.83)	89.16

*Note*. CI = confidence interval.

^a^RR = risk ratio

* Adjusted for urban-rural areas using CMH.

### Cause-specific preventable maternal death in different geographical regions

As shown in Table 4, cause-specific PMMR varied largely across the country during the 1996-2005 period, with obstetric haemorrhage being the leading cause followed by pregnancy-induced hypertension. Obstetric haemorrhage contributed to more than 50% of the preventable maternal deaths. During the entire study period, a significant decreasing trend was observed for obstetric haemorrhage-specific PMMR in the inland and remote areas, pregnancy-induced hypertension-specific PMMR in coastal regions and inlands, cardiac disorder-specific PMMR in coastal regions, and puerperal infection-specific PMMR in remote areas. When compared with the 1996-2000 period, there was a large reduction (44.22%) of obstetric haemorrhage-specific PMMR in remote areas during the years 2001-2005. The remote over coastal RR for obstetric haemorrhage were 7.84 (95% CI: 6.10-10.08) and 4.75 (95% CI:3.57-6.32), respectively, and the inland over coastal RR for obstetric haemorrhage were 2.96 (95% CI:2.32-3.78) and 2.59 (95% CI:1.98-3.39), respectively.

**Table 4:** Regional disparities of cause-specific preventable maternal mortality in China's different regions, 1996-2000 and 2001-2005

Cause	Preventable proportion (%)			Cause-specific mortality (per 100 000 live births)			**Adjusted RR**^**a**^(95% CI)*		
			
	Coastal	Inland	Remote	Coastal	Inland	Remote	Coastal	Inland	Remote
1996-2000									
Hemorrhage	84(34.71)	317(48.40)	364(59.19)	7.37	25.62	71.73	1	2.96(2.32-3.78)	7.84(6.10-10.08)
Pregnancy-induced hypertension	35(14.46)	94(14.35)	82(13.33)	3.07	7.60	16.16	1	2.19(1.47-3.26)	4.58(3.00-6.98)
Amniotic fluid embolism	32(13.22)	41(6.26)	12(1.95)	2.81	3.31	2.36	1	1.07(0.66-1.71)	0.79(0.41-1.54)
Cardiac disorders	32(13.22)	63(9.62)	37(6.02)	2.81	5.09	7.29	1	1.60(1.04-2.46)	2.20(1.35-3.58)
Puerperal infection	4(1.65)	24(3.66)	43(6.99)	0.35	1.94	8.47	1	3.92(1.43-10.73)	15.88(6.01-41.92)
Hepatic diseases	13(5.37)	17(2.60)	12(1.95)	1.14	1.37	2.36	1	1.12(0.55-2.27)	1.59(0.71-3.53)
2001-2005									
Hemorrhage	72(51.80)	268(50.85)	196(54.14)	6.97	21.88**	40.01**	1	2.59(1.98-3.39)	4.75(3.57-6.32)
Pregnancy-induced hypertension	18(12.95)	70(13.28)	50(13.81)	1.74***	5.72***	10.21	1	2.72(1.61-4.60)	4.80(2.78-8.30)
Amniotic fluid embolism	14(10.07)	32(6.07)	16(4.42)	1.36	2.61	3.27	1	1.62(0.86-3.06)	1.96(0.95-4.07)
Cardiac disorders	6(4.32)	54(10.25)	31(8.56)	0.58***	4.41	6.33	1	6.44(2.73-15.20)	9.06(3.66-22.46)
Puerperal infection	1(0.72)	18(3.42)	17(4.70)	0.10	1.47	3.47***	1	6.41(1.03-39.82)	16.44(2.65-102.15)
Hepatic diseases	2(1.44)	10(1.90)	7(1.93)	0.19	0.82	1.43	1	2.69(0.53-13.52)	5.00(0.79-31.57)

*Note*. CI = confidence interval.

^a^RR = risk ratio

* Adjusted for urban-rural areas using CMH.

** *P *< 0.001

***P < 0.05

### Distribution of the factors related to preventable maternal deaths

Factors associated with preventable maternal deaths varied by geographic locations (Table 5). Deaths related to factors at medical institutions increased in the following order: remote, inland and coastal areas. Deaths related to individual/family factors decreased in the same order. In coastal and inland regions, more than 50% of preventable deaths were associated with the knowledge/skill of medical institutions. In remote areas, the most frequent factors associated with preventable deaths were improper individual/family knowledge/skills (46.11%), followed by improper knowledge/skills of medical institutions (29.61%) and the financial status of the family (10.66%). In coastal regions and inland, the lack of essential knowledge/skills by health professionals at lower than the county level seemed to be a dominant problem, particularly for those at the township and county levels (Table 5).

**Table 5:** The distribution of factors related to preventable maternal mortality in China (%)

Problems	Coastal	Inland	Remote	**χ**^**2**^	P-value
**For individual/family**	**143(37.54)**	**523(44.24)**	**651(66.70)**		
Knowledge/skill	108(28.35)	351(29.69)	450(46.11)	73.482	< 0.001
Attitude	26(6.83)	110(9.30)	97(9.94)	3.233	0.199
Resources	9(2.36)	62(5.25)	104 (10.66)	38.720	< 0.001
**For medical institutions**	**237(62.19)**	**635(53.74)**	**305(31.25)**		
Knowledge/skill	223(58.52)	591(50.01)	289(29.61)		
Village-level	21(5.51)	197(16.67)	100(10.25)	40.238	< 0.001
Township-level	73(19.16)	167(14.13)	73(7.48)	41.229	< 0.001
County-level	79(20.73)	160(13.54)	82(8.40)	39.340	< 0.001
Province-level	50(13.12)	67(5.67)	34(3.48)	45.832	< 0.001
Attitude	5(1.31)	23(1.95)	9(0.92)	3.967	0.138
Resources	6(1.57)	8(0.68)	4(0.41)	5.316	0.070
Management	3(0.79)	13(1.1)	3(0.31)	4.529	0.104
**For social departments**	**1(0.27)**	**24(2.02)**	**20(2.05)**		
Knowledge/skill	0(0.00)	14(1.18)	4(0.41)	7.757	0.021
Attitude	0(0.00)	2(0.17)	0(0.00)		0.642*
Management	0(0.00)	2(0.17)	11(1.13)		0.004*
Resources	1(0.27)	6(0.50)	5(0.50)		1*

**Total**	**381(100.00)**	**1182(100.00)**	**976(100.00)**		

* Fisher's Exact Test

## Discussion

Based on data retrieved from the NMMSS, our study found that 86.1% of maternal deaths in China between 1996 and 2005 were preventable. Both the proportion and the MMR of preventable deaths differed by regions: underdeveloped, remote areas had higher MMRs than developed, coastal regions; rural areas had higher MMRs than urban areas; and remote rural areas had the highest MMRs. The factors associated with preventable death also had regional variations, which might explain the regional disparities in PMMR. The epidemiological features of preventable maternal mortality in China have not been described in detail; hence, our findings will be of great value to the government's intervention in reducing maternal deaths.

MMR has been used as one of the key indicators evaluating the socioeconomic development of a country/region. The risk factors related to maternal deaths are usually controlled with interventions or environment improvement, which means that the reduction of MMR is, in essence, that of preventable maternal mortality. Therefore, the PMMR of a country/region reflects both the quality of its obstetric services and the quality of life of the women there. Many reports show that the proportion of maternal deaths that are preventable varies worldwide, namely, 37% in Japan[25], 40% in the United States [20], 44.4% in South Australia[26], 70.2% in Nigeria[27], and as high as 90% in Brazil[28]. Our study indicated that the proportion of preventable maternal deaths in China between 1996 and 2005 was compatible with those in developing countries.

Although the MMR in coastal regions, inner lands and remote areas all declined during the 1996-2005 period, significant disparities were observed in urban/rural areas and by geographic locations. The PMMRs in remote and inlands areas were four and more than two times as high as that of coastal regions, respectively. It is noteworthy that the RR in inner lands was higher during the 2001-2005 period than during the 1996-2000 period. This suggests that the gap between inland and coastal regions in preventable maternal mortality might have been widening. Despite the high-speed economic growth in the past two decades, the country has seen an increasing disequilibrium in regional development. Official data released in 2005 on national economic growth showed that the average income per capita for coastal regions was 1.5 and 1.7 times higher than that for inner lands and remote areas, respectively, and was 3.5 times higher for urban areas than for rural areas. The differences were noticeably larger than that in 1996 [29]. Remote areas have lagged behind in economic development, but favourable state policies and health projects have enabled them to improve the health of local women and children by a larger margin and at a higher speed than inner lands [13]. By contrast, inner lands experienced a "bottleneck" in economic and medical undertakings because they did not have the economic advantages of the coastal regions or the favourable policies and projects of the remote areas. This situation was best reflected by the large difference in PMMR and relatively stable RR during different periods. In remote areas, the marked reduction in PMMR and minimization of RR during different periods further proved that special intervention programs such as "Reducing Maternal Mortality and Eliminating Newborn Tetanus"[30] by the government achieved a good result.

The cause-specific PMMR also presented regional differences in China during 1996-2005. Obstetric haemorrhage was the leading cause of maternal mortality across the country but comprised different proportions of the preventable cause-specific deaths; it was most prevalent in remote areas, followed by inner lands and coastal regions. As medical sciences advance, 90% of obstetric haemorrhage should be preventable when sufficient blood supply, easy transport, and skilful medical procedures are available [20]. Although the proportion of deaths due to obstetric haemorrhage increased in coastal regions during the 2001-2005 period, the MMR still remained the lowest among the three regions. In the United States and Europe, maternal deaths due to obstetric haemorrhage have been reduced to an extremely low level, and haemorrhage has been replaced by embolism, cardiac disorders, and other unavoidable factors as the leading cause of maternal mortality [31]. However, in developing countries such as China, the largest proportion of maternal deaths still results from obstetric haemorrhage. According to a WHO estimate, obstetric haemorrhage accounted for 30.8% of all maternal deaths among Asian women [32]. The situation is becoming worse in China, particularly in remote areas where women have the highest risk of dying from obstetric haemorrhage. Although there was a decreasing trend in other cause-specific PMMRs besides obstetric haemorrhage, regional gaps in the PMMR were still obvious. Women in remote and inland areas had a higher risk of dying from pregnancy-induced hypertension, cardiac disorders, and puerperal infection than those in coastal regions. To some extent, the reduction of MMR in China depends on the reduction of deaths due to these conditions.

It has been widely accepted that MMR in a specified area is affected by socioeconomic status in addition to the development of healthcare systems. In China, 80% of health resources were concentrated in big cities [33], and 80% of these resources were concentrated in large urban hospitals [34]. There were more abundant healthcare resources and human resources in coastal regions, and the number of technicians (with bachelor degrees and senior professional titles) was much higher than in inland and remote regions. In terms of types of obstetric services, such as caesarean section, hysterectomy and blood transfusion, there were no large differences between health institutions at the county level and provincial level. However, grassroots health institutions in rural and remote regions had the lowest obstetric service quality due to the inadequate medical facilities and obstetrician skill [34,35]. Undoubtedly, the coastal region of China has a well-developed socioeconomic and healthcare system. If effective measures are taken, the maternal healthcare in the inland and remote areas can achieve the same level.

Our findings reveal that the factors associated with maternal mortality also differed by region. Knowledge/skill of individual/family seemed to be the major factor for remote areas, where women or their families were usually under-educated or ill-informed, resulting in ignorance of pregnancy/delivery-related risks, absence of prenatal examination, or inability to identify risks of pregnancy-related complications. Most of these women underwent home delivery attended by inadequately trained family members or relatives [36]. As they were undereducated, elderly and slow at learning new knowledge, most midwives could not properly handle obstetric complications during delivery, such as retention of the placenta and uterine inertia, which may lead to maternal death at home or on the way to the hospital. In 2005, the hospital delivery rates in rural and remote areas were 76.22% and 33.20%, respectively, which is far lower than that in urban areas (95%) [37]. However, in coastal regions that enjoyed developed economies, rich health resources, easy transport and high accessibility of health services, most women preferred hospital delivery. The insufficient knowledge/skills of the health professionals became the major factor. The deaths happened mainly in medical institutions at the township and county level and usually resulted from such conditions such as delay in identifying or handling problems or risks during pregnancy, delivery, or puerperium; ignorance of proper referral for certain diseases; and poor surgical skills. Interestingly, remote areas had fewer problems than coastal regions concerning the knowledge/skills of health professionals, which was probably due to fewer hospital visits of the women in remote areas where health resources were scarce and access to obstetric services was inconvenient. The large proportion of maternal deaths due to individual/family factors may mask the significance of the knowledge/skills of medical institutions in remote areas. Thus, for the Chinese government, it is important to improve the knowledge/skills of individuals/families and health professionals in medical institutions, irrespective of where they live.

In summary, there are great regional disparities in preventable maternal mortality and associated factors. The decreasing trends observed in the overall PMMRs and cause-specific PMMRs suggest that the MMR in rural and inland regions can be reduced remarkably if effective measures are taken. Improvements in the economy or healthcare will be of great benefit to control or diminish environmental factors associated with preventable deaths, eventually minimizing MMR and the regional gaps within China. These findings also indicate that maternal mortality in a developing country/region will be reduced substantially if its economy, culture and healthcare are improved.

Our study has some limitations: (1) The early abortion-related maternal deaths outside the hospital were likely to be underreported, especially in the regions with a weak maternal and child healthcare system, although NMMSS covers the entire period of pregnancy and has unified quality control measures. (2) Direct analysis of the relationship between maternal death and prenatal healthcare was not possible because the detailed perinatal healthcare data for individual deaths were not collected. Many studies have shown that maternal death is associated with several social factors besides healthcare service quality [19,38]. Scholars have been aware of the large rural-urban difference and regional difference in maternal death for a long time; however, appropriate data to explore the cause of the difference cannot be found. This paper used the large population-based surveillance data, and therefore, the results were reliable, which was of great value for adopting effective measures to reduce maternal death in developing countries where developed, developing and underdeveloped regions coexisted.

## Conclusions

Most maternal mortality in China is preventable. Less developed regions, have a higher proportion of preventable maternal mortality and a higher risk of dying from pregnancy-related causes. The prevention of maternal mortality in inland and remote regions should be the focus of future work. We propose that the government focus its efforts on the following aspects in future interventions: The first is to improve basic antenatal care service, guaranteeing that each pregnant woman will receive at least five high-quality prenatal examinations, and to subsidize hospital delivery; the second is to establish an emergency rescue system for critically ill pregnant women, develop and improve referral systems among health institutions at township/county/provincial levels and create a green channel for critically ill pregnant women; the third is to strengthen health education, thus enhancing women's self-healthcare awareness and their ability to identify the risk of pregnancy complications and seek healthcare service, to promote professional training of obstetricians in basic-level medical units, specifically, to focus the county-level training on the prevention and treatment of obstetric haemorrhage and emergency skills for treatment of critically ill pregnant women, and to increase the skills of midwives during normal delivery and identify high-risk pregnant women in the township-level training.

## Competing interests

The authors declare that they have no competing interests.

## Authors' contributions

JL and LD made equal contributions, jointly designing the study and supervising all aspects of its implementation. XHL, QL and MRL participated in data collection, data analysis, data quality checking and results interpretation. WYZ, HW and RZ participated in the maternal death review at the state level. YPW and JZ led the writing, and JL and LD made critical comments on the drafts. All authors read and approved the final version.

## Pre-publication history

The pre-publication history for this paper can be accessed here:

http://www.biomedcentral.com/1471-2458/11/243/prepub

## References

[B1] United Nations Implementation of the United Nations Millennium Declaratio 2002 Report of the Secretary General

[B2] WHO, UNICEF, UNFPA Maternal mortality in 2000: estimates developed by WHO, UNICEF, UNFP 2004 Geneva: WHO

[B3] Hill K Thomas K AbouZahr C Walker N Say L Inoue M Suzuki E Estimates of maternal mortality worldwide between 1990 and 2005: an assessment of available data Lancet 2007 370 1311 1319 10.1016/S0140-6736(07)61572-4 17933645

[B4] Hogan MC Foreman KJ Naghavi M Ahn SY Wang M Makela SM Lopez AD Lozano R Murray CJL Maternal mortality for 181 countries, 1980-2008: a systematic analysis of progress towards Millennium Development Goal 5 Lancet 2010 375 1609 1623 10.1016/S0140-6736(10)60518-1 20382417

[B5] Kilpatrick SJ Crabtree KE Kemp A Geller S Preventability of maternal deaths: comparison between Zambian and American referral hospitals Obstet Gynecol 2002 100 321 326 10.1016/S0029-7844(02)02065-3 12151157

[B6] Sibbald B The struggle to reduce maternal mortality CMAJ 2007 177 243 245 17664441 10.1503/cmaj.070891 PMC1930195

[B7] Lang CT King JC Maternal mortality in the United States Best Pract Res Clin Obstet Gynaecol 2008 22 517 531 10.1016/j.bpobgyn.2007.10.004 18182328

[B8] Razum O Jahn A Blettner M Reitmaier P Trends in maternal mortality ratio among women of German and non-German nationality in West Germany, 1980-1996 Int J Epidemiol 1999 28 919 924 10.1093/ije/28.5.919 10597992

[B9] Schutte J Steegers E Schuitemaker N Santema J de Boer K Pel M Vermeulen G Visser W van Roosmalen J the Netherlands Maternal Mortality Committee Rise in maternal mortality in the Netherlands BJOG 2010 117 399 406 10.1111/j.1471-0528.2009.02382.x 19943828

[B10] Gonzalez R Requejo JH Nien JK Merialdi M Bustreo F Betran AP Tackling health inequities in Chile: maternal, newborn, infant, and child mortality between 1990 and 2004 Am J Public Health 2009 99 1220 1226 10.2105/AJPH.2008.143578 19443831 PMC2696659

[B11] Ronsmans C Graham WJ Maternal mortality: who, when, where, and why Lancet 2006 368 1189 200 10.1016/S0140-6736(06)69380-X 17011946

[B12] UNICEF, WHO, UNFPA Joint review of the maternal and child survival strategy in China 2006 Beijing: UNICEF

[B13] Gao J Qian J Tang S Eriksson B Blas E Health equity in transition from planned to market economy in China Health Policy Plan 2002 17 suppl 1 20 29 10.1093/heapol/17.suppl_1.20 12477738

[B14] Rudan I Chan KY Zhang JSF Theodoratou E Feng XL Salomon JA Lawn JE Cousens S Black RE Guo Y Campbell H Causes of deaths in children younger than 5 years in China in 2008 Lancet 2010 375 1083 1089 10.1016/S0140-6736(10)60060-8 20346815

[B15] Wang YP Miao L Dai L He CH Li XH Li MR Zhou GX Zhu J Liang J A study on ruraleurban differences in neonatal mortality rate in China, 1996-2006 J Epidemiol Community Health 2010 64 935 936 10.1136/jech.2009.090910 20584731

[B16] Rao K Chen Y Tian M Research on the classification of health status in China Chinese Journal of Health Statistics 1989 6 12 17 [In Chinese]

[B17] Lin LM Liu YL Mi J Liu JJ Feng SY Zhang S A survey of deaths of children under the age of 5 years in China Chin J Pediatr 1994 32 149 152 [In Chinese]

[B18] Ministry of Health China Health Statistics Yearbook 2005 2006 Beijing: Ministry of Health, PRC [In Chinese]

[B19] Li J Luo C Deng R Jacoby P de Klerk N Maternal mortality in Yunnan, China: recent trends and associated factors BJOG 2007 114 865 874 10.1111/j.1471-0528.2007.01362.x 17506792

[B20] Berg CJ Harper MA Atkinson SM Bell EA Brown HL Hage ML Mitra AG Moise KJ Callaghan WM Preventability of pregnancy-related deaths, results of a state-wide review Obstet Gynecol 2005 106 1228 1234 10.1097/01.AOG.0000187894.71913.e8 16319245

[B21] World Health Organization Beyond the numbers: reviewing maternal deaths and complications to make pregnancy safer 2004 Geneva: WHO 10.1093/bmb/ldg009 14711752

[B22] World Health Organization International Statistical Classification of Diseases and Related Health Problem (ICD-10) 1993 Geneva, Switzerland: WHO 10th rev

[B23] Ruan RS Epidemiology Principles and Methods 2004 1 Chengdu: Sichuan University Press

[B24] Agresti A Categorical Data Analysis 1990 New York: John Wiley and Sons, Inc

[B25] Nagaya K Fetters MD Ishikawa M Kabo T Koyanagi T Saito Y Causes of maternal mortality in Japan JAMA 2000 283 2661 2667 10.1001/jama.283.20.2661 10819948

[B26] De Lange TE Budde MP Heard AR Tucker G Kennare R Dekker GA Avoidable risk factors in perinatal deaths: A perinatal audit in South Australia Aust N Z J Obstet Gynaecol 2008 48 50 57 10.1111/j.1479-828X.2007.00801.x 18275572

[B27] Ozumba BC Nwogu-Ikojo EE Avoidable maternal mortality in Enugu, Nigeria Public Health 2008 122 354 360 10.1016/j.puhe.2007.04.018 17959207

[B28] Alves SV Maternal mortality in pernambuco, Brazil: what has changed in ten years? Reprod Health Matters 2007 15 134 144 10.1016/S0968-8080(07)30326-1 17938078

[B29] State Statistics Bureau China Statistical Yearbook 2005 2006 Beijing: State Statistics Bureau, PRC [In Chinese]

[B30] Liang J Zhu J Li MR Wang YP Dai L He CH Li Q Analysis of epidemiological characteristics of maternal mortality in target counties in 2007 Mod Prev Med 2009 36 1851 1860 [In Chinese]

[B31] Bodker B Hvidman L Weber T Moller M Aarre A Nielsen KM Sorensen JL Maternal deaths in Denmark 2002-2006 Acta Obstet Gynecol Scand 2009 88 556 562 10.1080/00016340902897992 19353337

[B32] Khan KS Wojdyla D Say L Gulmezoglu AM Van Look PFA WHO analysis of causes of maternal death: a systematic review Lancet 2006 367 1066 1074 10.1016/S0140-6736(06)68397-9 16581405

[B33] Haixia Lu Rational thoughts on imbalanced allocation of China's rural basic health resources Chinese Health Economics 2009 28 38 42 [In Chinese]

[B34] Rong Luo Qi Yang Xi Jin Analysis on medical and health personnel collocation of WCH of province and prefecture and county Maternal & Child health care of China 2007 22 2313 2315 [In Chinese]

[B35] Huang A Xi J Luo R Mu T Xiang M Wang C Pan X The situation of human resources in the county level maternal and children health care institutes Chin J Women Child Health 2010 11 130 133 [In Chinese]

[B36] Liang J Wang YP Wu YQ Zhou GX Zhun J Dai L Miao L Maternal mortality in rural areas in China Journal of Sichuan University (Medical Science Edition) 2004 35 258 60 [In Chinese] 15071934

[B37] Fuqiang Cui Purhati Hadler Stephen Xiaofeng Liang Analysis on new born hepatitis B immunization coverage and pregnant women hospital delivery rate in different regions Chinese journal of vaccines and immunization 2007 13 1 3 [In Chinese]

[B38] Zhu L Qin M Du L Jia W Yang Q Walker M Wen S Comparison of maternal mortality between migrating population and permanent residents in Shanghai, China, 1996-2005 BJOG 2009 116 401 407 10.1111/j.1471-0528.2008.01979.x 19187372

